# Supplementary material for: Quantifying the benefit offered by transcript assembly with Scallop-LR on single-molecule long reads
Source: Genome Biol. 2019 Dec 18;20:287. doi: 10.1186/s13059-019-1883-0 (PMC6918626; doi:10.1186/s13059-019-1883-0)
Supplement: Supplementary file 1 — Additional file 1 Supplementary materials, Tables S1-S13, Figures S1-S3 [file 13059_2019_1883_MOESM1_ESM.pdf]

Supplementary Materials

Quantifying the benefit offered by transcript assembly with Scallop-LR on single-molecule long reads

Laura H. Tung[1,2], Mingfu Shao[3], and Carl Kingsford[1]

[1]Computational Biology Department, School of Computer Science, Carnegie Mellon University
[2]Joint Carnegie Mellon University-University of Pittsburgh Ph.D. Program in Computational Biology
[3]Department of Computer Science and Engineering, The Pennsylvania State University

1. Merging multiple SRA Runs from the same BioSample into one dataset

In most of the PacBio datasets in SRA, one BioSample has multiple SRA Runs. We merge multiple SRA Runs that belong to the same BioSample into one dataset. PacBio sequencing uses the template called SMRTbell that is a closed, single-stranded circular DNA created by ligating adaptors to both ends of a target double-stranded cDNA molecule. The sequencing is based on a SMRT Cell, a chip with consumable substrates comprising arrays of zero-mode waveguide (ZMW) nanostructures, and a SMRTbell diffuses into a sequencing unit ZMW on it. The real-time observation of a SMRT Cell is called a movie, and an SRA Run usually contains one movie and sometimes contains multiple movies. One BioSample has multiple SRA Runs because the experimenters used multiple movies (i.e. multiple SMRT Cells) to increase the coverage so that those low-abundance, long isoforms can be captured in Iso-Seq Analysis, since the "polish" step in Iso-Seq Analysis keeps only the isoforms with at least two full-length reads to support them. In most cases, the experimenters also used a size selection sequencing strategy, that is, isoforms that are in different size ranges are split into separate independent SMRTbell libraries for sequencing, so that larger isoforms are not detrimentally dominated by smaller isoform molecules during the sequencing. Thus, different SRA Runs are designated for different size ranges. Therefore, we use one BioSample instead of one SRA Run to represent one dataset in our analysis, and we merge multiple SRA Runs into that dataset.

2. Software versions and options used in the analysis workflow

The software versions and options used in the analysis workflow are summarized in the following:

Iso-Seq Analysis: Iso-Seq2 from SMRT Link v5.1.0.
Minimap2: v2.2. Options: *-ax splice*.
StringTie: v1.3.2d. Options: *-c 1.0*.
Scallop-LR: v0.9.1. Options: *-c <ccs_read_info > --min_num_hits_in_bundle 1*.
Gffcompare: v0.9.9c. Options: *-M -N -r <reference_annotation>*.
SQANTI: v1.2. Options: *-g*.
rnaQUAST: v.1.5.1. Options: *--transcripts <multiple_assemblies> --reference <reference_genome> --gene_db <gene_database> --gmap_index <gmap_index> --labels*

<labels> --no_plots --disable_infer_genes --disable_infer_transcripts --lower_threshold <lower_threshold> --upper_threshold <upper_threshold>.

Transrate: v1.0.3. Options: *--assembly <assembly> --reference <reference_transcriptome>.*

3. Assessment of predicted transcripts that partially match known transcripts in mouse data

Figures S1, S2, and S3 show box-whisker plots of matched transcripts in matched fraction bins, assembled isoforms in assembled fraction bins, "mean isoform assembly" and "mean fraction of transcript matched" for Scallop-LR, StringTie, and Iso-Seq Analysis on the eight mouse datasets based on rnaQUAST evaluations. Full results are shown in Tables S8.1-S8.8.

In the mouse data, Scallop-LR predicts more transcripts that have a high fraction of their bases matching reference transcripts than Iso-Seq Analysis. From Tables S8.1-S8.8, in the high % bins of the “x-y% matched transcripts” (75-95% and 95-100% matched), Scallop-LR consistently has more x-y% matched transcripts than Iso-Seq Analysis. However, unlike in the human data, Scallop-LR consistently has fewer x-y% matched transcripts than StringTie in the high % bins. These trends are visualized in Figure S1 (75-95% and 95-100% matched bins).

However, on average, Scallop-LR transcripts match reference transcripts better than StringTie transcripts. In Tables S8.1-S8.8, Scallop-LR consistently has much higher values of “Mean fraction of transcript matched” than StringTie. Scallop-LR has slightly lower values than Iso-Seq Analysis though. These trends are visualized in Figure S3 (right: “Mean fraction of transcript matched”).

In the mouse data, there are more reference transcripts that have a high fraction of their bases being captured/covered by Scallop-LR transcripts than by Iso-Seq Analysis predicted transcripts. From Tables S8.1-S8.8, in the high % bins of the “x-y% assembled isoforms” (75-95% and 95-100% assembled), Scallop-LR consistently has more x-y% assembled isoforms than Iso-Seq Analysis. However, Scallop-LR consistently has fewer x-y% assembled isoforms than StringTie in the high % bins. These trends are visualized in Figure S2 (75-95% and 95-100% assembled bins).

However, on average, reference transcripts are better captured/covered by Scallop-LR transcripts than by StringTie transcripts and Iso-Seq Analysis transcripts. In Tables S8.1-S8.8, Scallop-LR consistently has higher values of “Mean isoform assembly” than both StringTie and Iso-Seq Analysis. Iso-Seq Analysis consistently has higher values than StringTie. This trend is visualized in Figure S3 (left: “Mean isoform assembly”).

The quality of StringTie transcripts in the mouse data is somewhat improved compared to that in the human data. As in the human data, StringTie consistently has significantly more unannotated transcripts than both Scallop-LR and Iso-Seq Analysis (Tables S8.1-S8.8). However, in Figure S1, unlike Figure 7, in the 0-50% matched bin StringTie no longer has a very high number of transcripts. This indicates that StringTie performs better in the mouse data than in the human data. In Figure S2, though, in the 0-50% assembled bin StringTie still has significantly higher numbers of isoforms than both Scallop-LR and Iso-Seq Analysis, similar to Figure 8.

4. Evaluation of Scallop-LR and StringTie on simulated human data

We evaluated Scallop-LR and StringTie on a simulated human dataset (Liu *et al.*, 2019). The transcriptome that was used to generate the simulated long reads originated from the Ensembl annotation *Homo sapiens* GRCh38.94 and was a subset of the transcripts in this Ensembl annotation, by removing unfinished scaffolds, transcripts shorter than 200 bp, annotations with an unknown reference, etc. and randomly selecting alternative-splicing genes, single-splicing genes, and genes with small exons (< 31bp). The PacBio PBSIM tool was used to generate the simulated CCS reads from this transcriptome. The simulation was model-based using the CCS model, and three runs of simulations were performed by using three different sequencing depths 4X, 10X and 30X respectively. We merged the CCS reads generated with the three sequencing depths together to obtain this simulated human dataset. We used the transcripts in the transcriptome sequences that were used to generate the simulated CCS reads to extract the transcripts' records and their corresponding genes' records from the Ensembl annotation *Homo sapiens* GRCh38.94 to obtain an annotation GTF file. This extracted annotation GTF file serves as the "ground truth" and contains 7810 multi-exon transcripts.

In the Gffcompare evaluation, the extracted annotation GTF file corresponding to the transcriptome that was used to generate the simulated CCS reads serves as the reference annotation. Scallop-LR demonstrates both higher sensitivity and higher precision than StringTie (Table S12), consistent with the trends on the real human datasets. Note that since the simulated CCS reads do not contain the primer information, Scallop-LR's transcript boundary identification algorithm through extracting the boundary information from long reads is not used on the simulated data.

In the rnaQUAST evaluation, the extracted annotation GTF file corresponding to the transcriptome that was used to generate the simulated CCS reads is used to make the gene annotation database. Therefore, the metric "x-y% assembled isoforms" is computed relative to the initial set of expressed isoforms that was used to generate the simulated reads, rather than all known isoforms. StringTie has more "x-y% assembled isoforms" in the 95-100% bin than Scallop-LR (Table S13), consistent with the trend on the majority of the real human datasets. However, Scallop-LR has more "x-y% assembled isoforms" in the 75-95% bin than StringTie, which is different from the majority of the real human datasets. The results of the simulated data confirm that StringTie assembles more "95-100% assembled isoforms" than Scallop-LR, but Scallop-LR assembles more correctly predicted transcripts than StringTie (Table S12). This implies that while Scallop-LR outperforms StringTie in terms of the exact reference-matching transcripts (100% assembled, correct predictions), StringTie transcripts cover more reference transcripts on 95-99% of their bases than Scallop-LR transcripts.

We further performed the rnaQUAST evaluation on the simulated dataset by using the entire Ensembl annotation (*Homo sapiens* GRCh38.94) as the reference annotation. The resulting "95-100% assembled isoforms" are 3094 and 4613 for Scallop-LR and StringTie respectively. Compared with the corresponding results in Table S13 (which uses the "ground truth" as the reference annotation), StringTie assembles 365 more "95-100% assembled isoforms" when the entire Ensembl annotation is used as the reference. This implies that StringTie assembles 365 transcripts that are not in the ground truth but appear to be misassembled to match the reference

transcriptome. For Scallop-LR, the number of “95-100% assembled isoforms” by using the entire Ensembl annotation as the reference is very close to the number of “95-100% assembled isoforms” by using the “ground truth” as the reference (the difference between the numbers of “95-100% assembled isoforms” by using these two references is -9). This result may suggest that, for the real datasets, the “95-100% assembled isoforms” of StringTie could be somewhat inflated (i.e. some assembled transcripts are not in the ground truth but are misassembled to match certain transcripts in the reference), as we do not know the ground truth for the real datasets and use all known isoforms as the reference when evaluating the real data. On the other hand, based on this evidence, it seems that Scallop-LR stays consistent on this measure when the entire Ensembl annotation or the “ground truth” is used as the reference.

Table S1: Human Data: Sensitivity, Precision, and PR-AUC of Scallop-LR, StringTie, and Iso-Seq Analysis

Datasets	Sensitivity (%)			Precision (%)			PR-AUC		
	Scallop-LR	StringTie	Iso-Seq	Scallop-LR	StringTie	Iso-Seq	Scallop-LR	StringTie	Iso-Seq
SAMN00001694	5.50	4.32	3.30	38.47	33.63	62.64	0.03389	0.02664	0.01868
SAMN00001695	5.36	4.39	3.33	42.96	36.05	60.76	0.03363	0.02821	0.01808
SAMN00001696	4.48	3.93	2.80	47.29	40.24	65.59	0.02916	0.02573	0.01636
SAMN00006465	5.32	4.57	3.70	46.28	40.10	63.54	0.03563	0.03048	0.02085
SAMN00006466	5.05	4.25	3.49	48.42	35.70	65.82	0.03489	0.02796	0.02051
SAMN00006467	4.62	3.96	3.09	50.57	36.71	68.43	0.03200	0.02640	0.01875
SAMN00006579	5.19	4.29	3.51	43.52	34.68	61.63	0.03359	0.02777	0.01916
SAMN00006580	4.87	4.09	3.26	45.89	32.98	63.91	0.03239	0.02648	0.01839
SAMN00006581	5.09	4.16	3.42	43.31	33.87	62.47	0.03287	0.02733	0.01906
SAMN08182059	5.29	4.12	3.09	36.34	34.34	54.52	0.03138	0.02452	0.01515
SAMN08182060	5.52	4.42	3.34	43.59	37.82	61.30	0.03617	0.02837	0.01832
SAMN04563763	4.87	4.01	3.65	46.90	41.37	62.94	0.03320	0.02600	0.02047
SAMN07611993	7.60 (7.26)	5.43	0.87	28.97 (46.47)	32.65	55.42	0.04057	0.02910	0.00427
SAMN04169050	6.86 (6.61)	4.83	4.62	30.90 (51.74)	34.61	55.52	0.03807	0.02815	0.02296
SAMN04251426.1	5.70 (5.46)	4.44	3.40	29.32 (40.72)	32.40	49.64	0.02738	0.02457	0.01479
SAMN04251426.2	5.76 (5.56)	4.44	3.49	29.92 (41.18)	32.39	49.19	0.02749	0.02478	0.01507
SAMN04251426.3	5.81 (5.52)	4.58	3.51	30.09 (40.37)	33.44	48.93	0.02806	0.02537	0.01509
SAMN04251426.4	5.78 (5.47)	4.55	3.52	30.59 (40.91)	33.82	49.05	0.02828	0.02606	0.01492

The above table compares the Gffcompare evaluation results for Scallop-LR, StringTie, and Iso-Seq Analysis on human data. 18 human PacBio datasets were extracted from SRA, each corresponding to one BioSample and named by the BioSample ID (except that the last four datasets are four replicates for one BioSample). Multiple SRA Runs that belong to each BioSample were merged into a large dataset to perform the analyses. The first nine datasets were sequenced using the RS instrument and the last nine datasets were sequenced using the RS II instrument. Sensitivity is the ratio of the number of correctly predicted known transcripts over the total number of known transcripts, and precision is the ratio of the number of correctly predicted known transcripts over the total number of predicted transcripts. PR-AUC was calculated from the precision-recall curves we generated. The values within the parentheses are the adjusted sensitivity and adjusted precision. The adjusted sensitivity for Scallop-LR was calculated by matching the precision of StringTie, and the adjusted precision for Scallop-LR was calculated by matching the sensitivity of StringTie. The adjusted sensitivity and precision were only calculated for the last six datasets, since the last six datasets have opposite trends on sensitivity and precision comparing Scallop-LR and StringTie.

Table S2: Human Data: Correctly Predicted Known Transcripts, Total Multi-Exon Transcripts, and Potential Novel Isoforms of Scallop-LR, StringTie, and Iso-Seq Analysis

Datasets	# Potential Novel Isoforms			# Total Multi-Exon Transcripts			# Correctly Predicted Known Transcripts		
	Scallop-LR	StringTie	Iso-Seq	Scallop-LR	StringTie	Iso-Seq	Scallop-LR	StringTie	Iso-Seq
SAMN00001694	12050	6827	2847	24903	22370	9166	9580	7522	5742
SAMN00001695	9905	6149	2856	21731	21201	9554	9336	7642	5805
SAMN00001696	7425	5129	2122	16476	16983	7423	7791	6834	4869
SAMN00006465	9112	6111	2941	20019	19847	10136	9264	7958	6440
SAMN00006466	8054	5387	2561	18171	20715	9236	8798	7396	6079
SAMN00006467	6838	4710	2054	15900	18783	7865	8040	6896	5382
SAMN00006579	10250	6020	3175	20742	21508	9906	9027	7460	6105
SAMN00006580	8623	5295	2640	18467	21607	8870	8474	7125	5669
SAMN00006581	10064	5736	2974	20458	21376	9531	8861	7241	5954
SAMN08182059	13318	7610	3609	25332	20893	9876	9206	7175	5384
SAMN08182060	10207	6732	2951	22038	20348	9478	9606	7696	5810
SAMN04563763	7496	5464	2965	18078	16857	10087	8478	6973	6349
SAMN07611993	22834	10532	852	45657	28953	2741	13226	9453	1519
SAMN04169050	22403	9059	5696	38657	24280	14493	11946	8403	8047
SAMN04251426.1	17074	8572	4887	33824	23863	11913	9916	7731	5914
SAMN04251426.2	16871	8403	5090	33534	23849	12363	10034	7724	6081
SAMN04251426.3	16916	8580	5191	33637	23850	12472	10122	7976	6103
SAMN04251426.4	16347	8314	5194	32908	23423	12476	10065	7922	6119

The above table compares additional Gffcompare evaluation results for Scallop-LR, StringTie, and Iso-Seq Analysis on human data. The same 18 human PacBio datasets as described in Table S1 were evaluated. A Correctly Predicted Known Transcript is a transcript that has the exact intron-chain matching with a transcript in the reference annotation. A Potential Novel Isoform is a predicted transcript that shares at least one splice junction with a reference transcript. The # Total Multi-Exon Transcripts is the total number of predicted multi-exon transcripts.

Table S3: Mouse Data: Sensitivity, Precision, and PR-AUC of Scallop-LR, StringTie, and Iso-Seq Analysis

Datasets	Sensitivity (%)			Precision (%)			PR-AUC		
	Scallop-LR	StringTie	Iso-Seq	Scallop-LR	StringTie	Iso-Seq	Scallop-LR	StringTie	Iso-Seq
SAMEA3374575	3.82	4.18 (3.70)	2.52	60.50	51.08 (57.55)	72.74	0.02538	0.02716	0.01571
SAMEA3374576	4.00	4.49 (3.87)	2.79	64.86	55.06 (62.48)	77.74	0.02880	0.03069	0.01937
SAMEA3374577	3.68	4.25 (3.61)	2.52	67.14	56.71 (65.44)	78.23	0.02668	0.02913	0.01744
SAMEA3374578	3.49	3.96 (3.38)	2.42	65.74	55.86 (64.21)	80.37	0.02553	0.02714	0.01695
SAMEA3374579	4.72	5.24 (4.48)	2.98	63.83	50.86 (58.13)	77.44	0.03338	0.03431	0.02040
SAMEA3374580	4.70	5.26 (4.52)	2.85	62.42	48.50 (58.28)	77.87	0.03385	0.03475	0.01933
SAMEA3374581	5.43	5.73 (4.93)	3.33	60.04	46.09 (50.63)	73.58	0.03838	0.03568	0.02155
SAMEA3374582	4.27	4.71 (4.05)	2.49	57.57	45.62 (53.16)	75.21	0.02932	0.02870	0.01637

This table compares the Gffcompare evaluation results for Scallop-LR, StringTie, and Iso-Seq Analysis on mouse data. Eight mouse PacBio datasets were extracted from SRA, each corresponding to one BioSample and named by the BioSample ID. Multiple SRA Runs that belong to each BioSample were merged into a large dataset to perform the analyses. All eight datasets were sequenced using the RS instrument. Sensitivity, precision, and PR-AUC are as described in Table S1. The values within the parentheses are the adjusted sensitivity and adjusted precision. The adjusted sensitivity for StringTie was calculated by matching the precision of Scallop-LR, and the adjusted precision for StringTie was calculated by matching the sensitivity of Scallop-LR. The adjusted sensitivity and precision were calculated for all eight datasets, since all eight datasets have opposite trends on sensitivity and precision comparing Scallop-LR and StringTie.

Table S4: Mouse Data: Correctly Predicted Known Transcripts, Total Multi-Exon Transcripts, and Potential Novel Isoforms of Scallop-LR, StringTie, and Iso-Seq Analysis

Datasets	# Potential Novel Isoforms			# Total Multi-Exon Transcripts			# Correctly Predicted Known Transcripts		
	Scallop-LR	StringTie	Iso-Seq	Scallop-LR	StringTie	Iso-Seq	Scallop-LR	StringTie	Iso-Seq
SAMEA3374575	1973	2476	829	6879	8913	3768	4162	4553	2741
SAMEA3374576	1671	2206	699	6707	8870	3903	4350	4884	3034
SAMEA3374577	1468	1973	596	5962	8155	3505	4003	4625	2742
SAMEA3374578	1434	1958	520	5774	7723	3280	3796	4314	2636
SAMEA3374579	2003	2973	663	8048	11221	4193	5137	5707	3247
SAMEA3374580	2020	3237	543	8204	11801	3981	5121	5724	3100
SAMEA3374581	2714	3806	832	9842	13531	4920	5909	6237	3620
SAMEA3374582	1803	2934	414	8069	11233	3606	4645	5125	2712

This table compares additional Gffcompare evaluation results for Scallop-LR, StringTie, and Iso-Seq Analysis on mouse data. The same eight mouse PacBio datasets as described in Table S3 were evaluated. The Correctly Predicted Known Transcript, Potential Novel Isoform, and # Total Multi-Exon Transcripts are as described in Table S2.

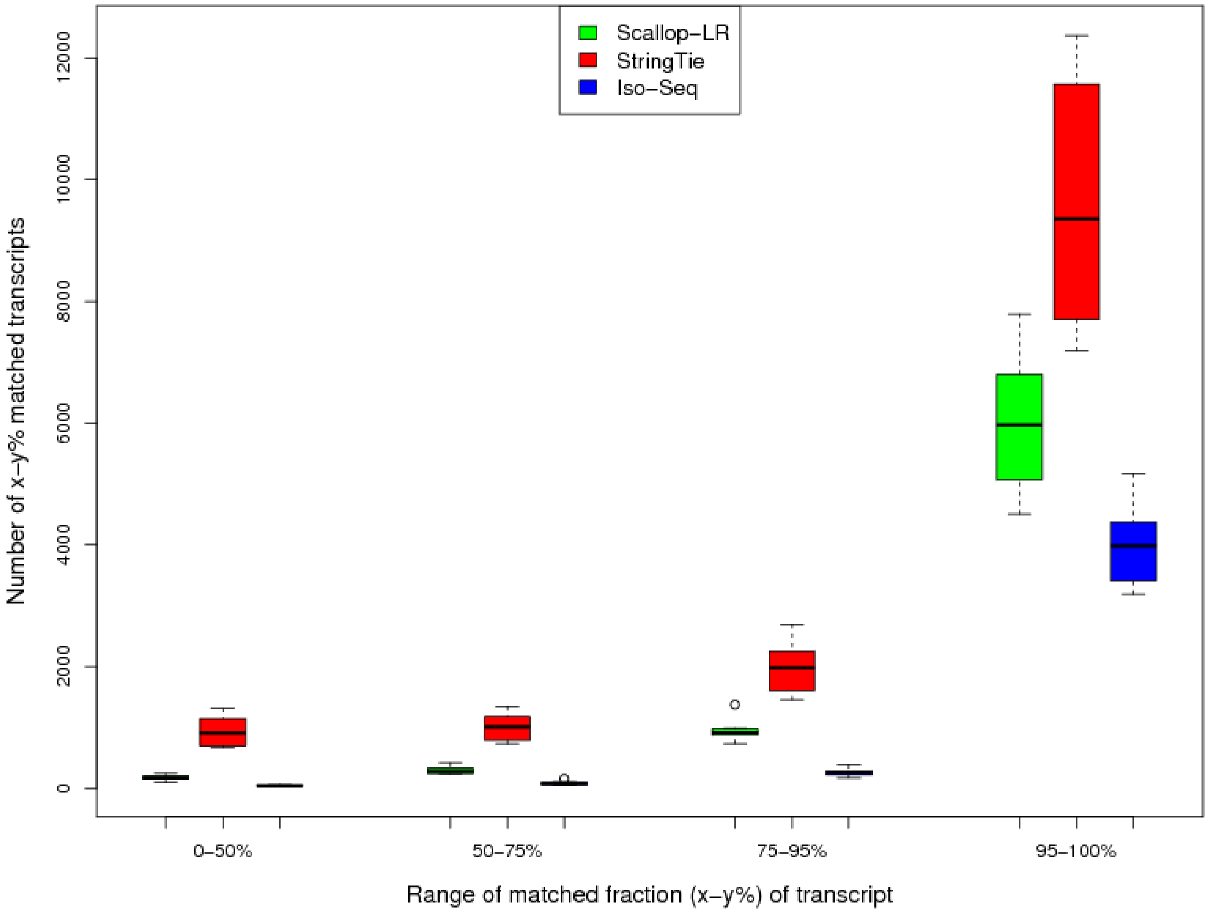

Figure S1: Mouse data: box-whisker plots of matched transcripts in four matched fraction bins for Scallop-LR, StringTie, and Iso-Seq Analysis, based on rnaQUAST evaluations. This figure is to compare numbers of x-y% matched transcripts. The same eight mouse PacBio datasets as described in Table S3 were evaluated via rnaQUAST. Figure axis descriptions are the same as in Figure 7.

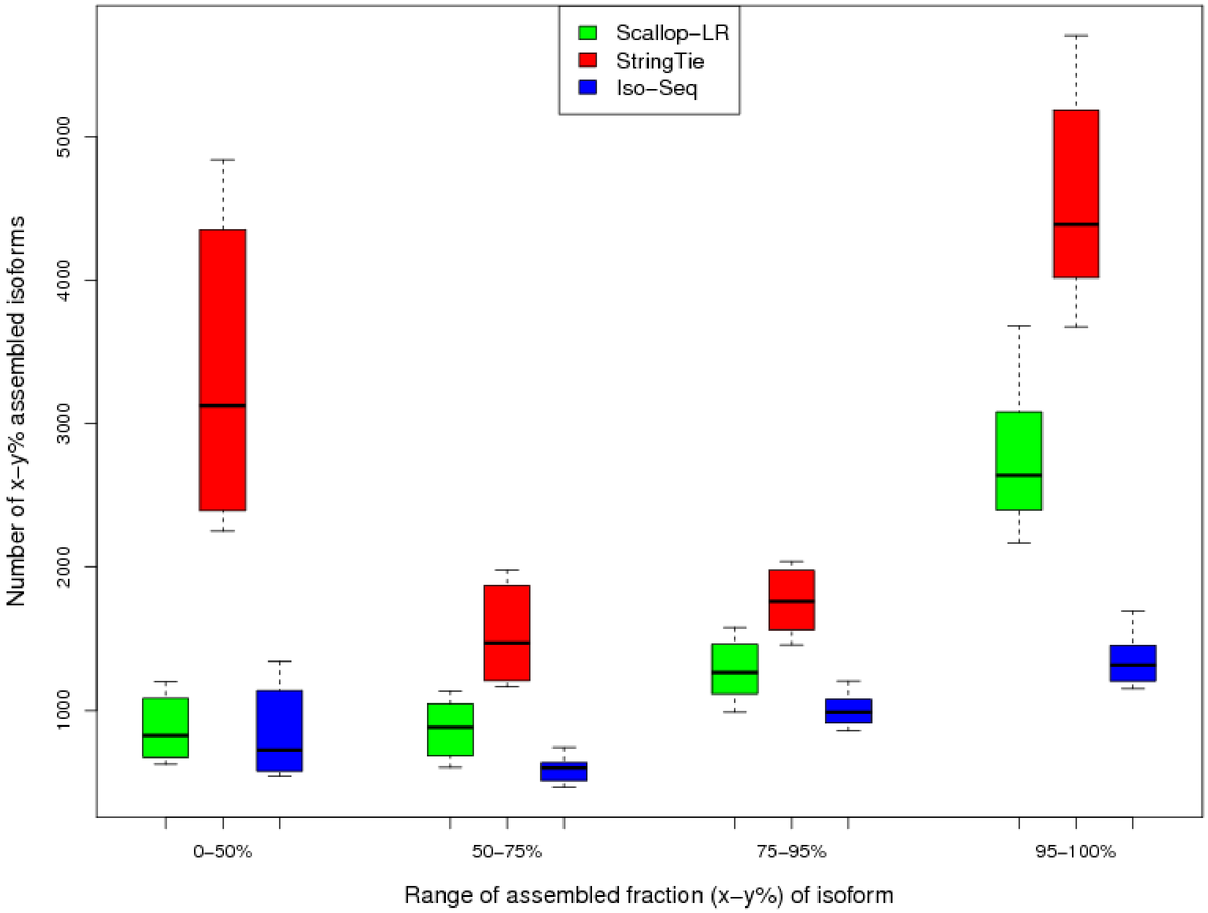

Figure S2: Mouse data: box-whisker plots of assembled isoforms in four assembled fraction bins for Scallop-LR, StringTie, and Iso-Seq Analysis, based on rnaQUAST evaluations. This figure is to compare numbers of x-y% assembled isoforms. The same eight mouse PacBio datasets as described in Table S3 were evaluated via rnaQUAST. Figure axis descriptions are the same as in Figure 8.

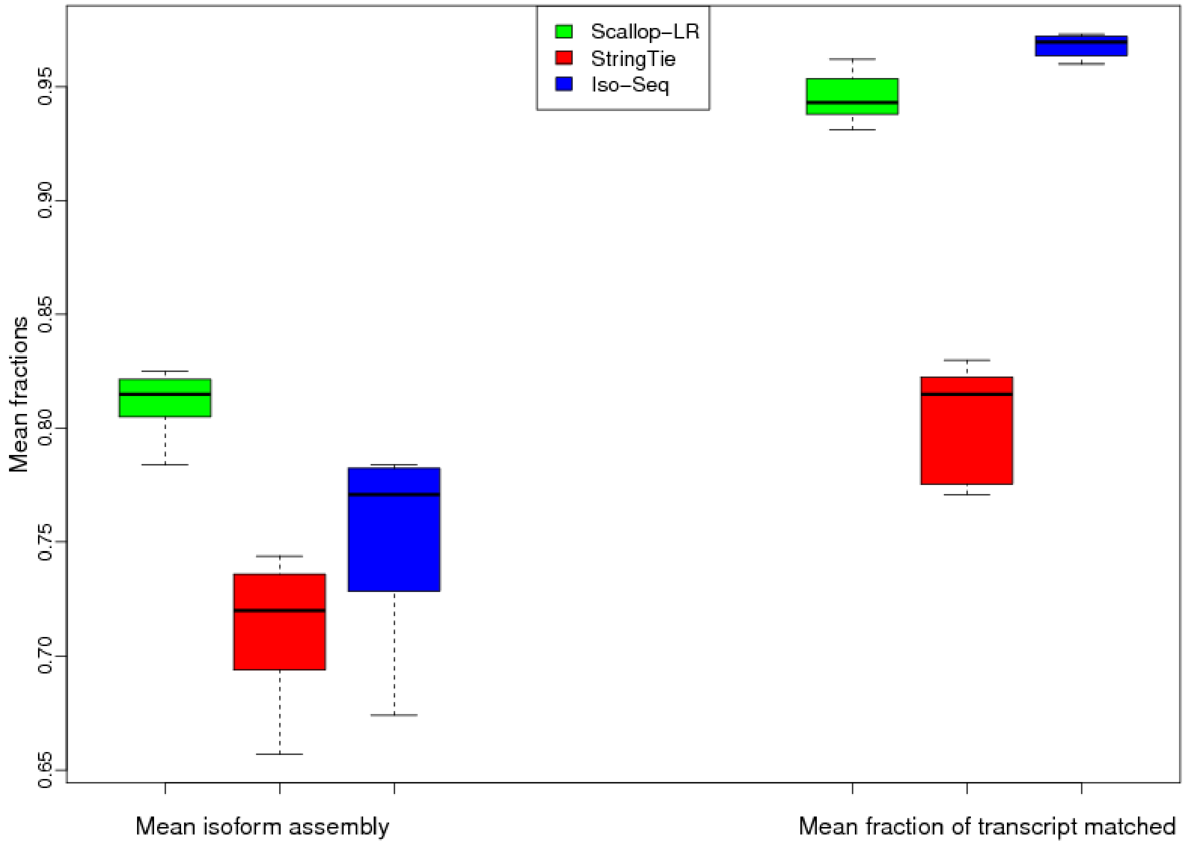

Figure S3: Mouse data: box-whisker plots of mean isoform assembly and mean fraction of transcript matched for Scallop-LR, StringTie, and Iso-Seq Analysis, based on rnaQUAST evaluations. The same eight mouse PacBio datasets as described in Table S3 were evaluated via rnaQUAST. Figure axis descriptions are the same as in Figure 9.

Table S5: Human Data: Numbers of FSM, NIC, NNC, and ISM transcripts of Scallop-LR and Iso-Seq Analysis based on SQANTI evaluations

Datasets	# of FSM		# of NIC		# of NNC		# of ISM	
	Scallop-LR	Iso-Seq	Scallop-LR	Iso-Seq	Scallop-LR	Iso-Seq	Scallop-LR	Iso-Seq
SAMN00001694	9594	5736	6375	1586	5579	1232	2411	487
SAMN00001695	9329	5800	5765	1624	4045	1263	1934	776
SAMN00001696	7787	4861	4054	1198	3294	930	934	369
SAMN00006465	9266	6434	5080	1638	3962	1281	1141	630
SAMN00006466	8796	6071	4548	1488	3479	1057	837	493
SAMN00006467	8039	5375	3890	1173	2890	854	633	340
SAMN00006579	9026	6096	5979	1759	4115	1297	903	505
SAMN00006580	8472	5665	4903	1478	3628	1060	772	415
SAMN00006581	8859	5947	5889	1765	4104	1176	919	470
SAMN08182059	9220	5545	7874	2206	5488	1611	1898	1612
SAMN08182060	9616	5941	4882	1433	5375	1674	1244	888
SAMN04563763	8480	6535	4174	1899	3368	1297	1556	1066
SAMN07611993	13275	1564	13406	533	9518	438	7186	531
SAMN04169050	11984	8193	12490	3364	9737	2616	2616	1141
SAMN04251426.1	9934	6114	9831	3497	7174	2175	5070	1729
SAMN04251426.2	10058	6317	9771	3630	6972	2248	4813	1886
SAMN04251426.3	10150	6327	9830	3814	6970	2249	4969	1840
SAMN04251426.4	10081	6340	9545	3719	6695	2282	4818	1851

The above table compares the SQANTI evaluation results for Scallop-LR and Iso-Seq Analysis on human data. The same 18 human PacBio datasets as described in Table S1 were evaluated. FSM (Full Splice Match): the predicted transcript that matches a reference transcript at all splice junctions. ISM (Incomplete Splice Match): the predicted transcript that matches consecutive, but not all, splice junctions of a reference transcript. NIC (Novel in Catalog): the predicted transcript that contains new combinations of already annotated splice junctions or novel splice junctions formed from already annotated donors and acceptors. NNC (Novel Not in Catalog): the predicted transcript that contains novel splice junctions formed from novel donors or/and novel acceptors.

Table S6: Mouse Data: Numbers of FSM, NIC, NNC, and ISM transcripts of Scallop-LR and Iso-Seq Analysis based on SQANTI evaluations

Datasets	# of FSM		# of NIC		# of NNC		# of ISM	
	Scallop-LR	Iso-Seq	Scallop-LR	Iso-Seq	Scallop-LR	Iso-Seq	Scallop-LR	Iso-Seq
SAMEA3374575	4170	2865	503	153	1366	610	513	258
SAMEA3374576	4358	3145	503	207	1125	470	489	240
SAMEA3374577	4009	2840	446	169	983	378	355	194
SAMEA3374578	3799	2718	545	202	906	326	362	188
SAMEA3374579	5137	3410	591	190	1400	463	689	437
SAMEA3374580	5121	3284	736	210	1323	359	843	714
SAMEA3374581	5912	3834	931	283	1784	522	936	905
SAMEA3374582	4644	2874	685	199	1198	254	1398	1261

This table compares the SQANTI evaluation results for Scallop-LR and Iso-Seq Analysis on mouse data. The same eight mouse PacBio datasets as described in Table S3 were evaluated. Metrics descriptions are the same as in Table S5.

Table S7.1: rnaQUAST evaluation results for human dataset SAMN08182059, comparing Scallop-LR, StringTie, and Iso-Seq Analysis

Metrics	Scallop-LR	StringTie	Iso-Seq
# Transcripts	25364	32230	11467
Aligned	25363	32224	11467
Uniquely aligned	25361	32028	11444
Unaligned	1	6	0
Misassemblies	2	4	4
0-50% assembled isoforms	1412	4156	1681
50-75% assembled isoforms	1827	2332	1358
75-95% assembled isoforms	2994	3012	1994
95-100% assembled isoforms	6052	6691	2507
Mean isoform assembly	0.834	0.729	0.74
0-50% matched transcripts	1917	4466	267
50-75% matched transcripts	2057	2394	231
75-95% matched transcripts	4612	3659	734
95-100% matched transcripts	16595	12570	9971
Unannotated	180	9118	260
Mean fraction of transcript matched	0.883	0.558	0.939

This table compares the rnaQUAST evaluation results for Scallop-LR, StringTie, and Iso-Seq Analysis on a human dataset. “Isoforms” refer to reference transcripts from the gene annotation database, and “transcripts” refer to predicted transcripts. “# Transcripts” is the total number of predicted transcripts (including single-exon transcripts). “Aligned” is the number of transcripts which have at least one significant alignment to the reference genome. “Uniquely aligned” is the number of transcripts which have a single significant alignment. “Unaligned” is the number of transcripts without any significant alignments. “Misassemblies” are the transcripts that have discordant best-scored alignments (i.e. partial alignments that are mapped to different strands, different chromosomes, in reverse order, or too far away). “Unannotated” is the number of transcripts that do not cover any isoform from the annotation database. “x-y% assembled isoforms” is the number of isoforms from the annotation database that have at least x% and at most y% captured by a single predicted transcript. “x-y% matched transcripts” is the number of transcripts that have at least x% and at most y% matching an isoform from the annotation database. “Mean isoform assembly” is the average value of assembled fractions, where the assembled fraction of an isoform is computed as the largest number of its bases captured by a single predicted transcript divided by its length. “Mean fraction of transcript matched” is the average value of matched fractions, where the matched fraction of a transcript is computed as the number of its bases covering an isoform divided by the transcript length.

Table S7.2: rnaQUAST evaluation results for human dataset SAMN08182060, comparing Scallop-LR, StringTie, and Iso-Seq Analysis

Metrics	Scallop-LR	StringTie	Iso-Seq
# Transcripts	22065	29664	10233
Aligned	22065	29660	10233
Uniquely aligned	22057	29480	10219
Unaligned	0	4	0
Misassemblies	3	4	2
0-50% assembled isoforms	1205	3659	1154
50-75% assembled isoforms	1547	1994	1236
75-95% assembled isoforms	2759	2810	2035
95-100% assembled isoforms	6760	7420	3064
Mean isoform assembly	0.855	0.757	0.794
0-50% matched transcripts	1677	4263	266
50-75% matched transcripts	1806	2147	186
75-95% matched transcripts	4035	3588	652
95-100% matched transcripts	14376	11914	9004
Unannotated	165	7726	123
Mean fraction of transcript matched	0.881	0.577	0.948

This table compares the rnaQUAST evaluation results for Scallop-LR, StringTie, and Iso-Seq Analysis on a human dataset. Metrics descriptions are the same as in Table S7.1.

Table S7.3: rnaQUAST evaluation results for human dataset SAMN04563763, comparing Scallop-LR, StringTie, and Iso-Seq Analysis

Metrics	Scallop-LR	StringTie	Iso-Seq
# Transcripts	18097	29003	11103
Aligned	18096	29002	11103
Uniquely aligned	18093	28788	11088
Unaligned	1	1	0
Misassemblies	2	3	0
0-50% assembled isoforms	2212	5762	1926
50-75% assembled isoforms	1792	2496	1411
75-95% assembled isoforms	2034	2227	1954
95-100% assembled isoforms	4523	5567	2669
Mean isoform assembly	0.765	0.657	0.731
0-50% matched transcripts	927	3126	362
50-75% matched transcripts	1657	2337	327
75-95% matched transcripts	4318	3769	1166
95-100% matched transcripts	11053	11733	9105
Unannotated	138	8017	143
Mean fraction of transcript matched	0.89	0.59	0.934

This table compares the rnaQUAST evaluation results for Scallop-LR, StringTie, and Iso-Seq Analysis on a human dataset. Metrics descriptions are the same as in Table S7.1.

Table S7.4: rnaQUAST evaluation results for human dataset SAMN07611993, comparing Scallop-LR, StringTie, and Iso-Seq Analysis

Metrics	Scallop-LR	StringTie	Iso-Seq
# Transcripts	45773	42040	3382
Aligned	45769	42028	3382
Uniquely aligned	45763	41813	3378
Unaligned	4	12	0
Misassemblies	5	9	3
0-50% assembled isoforms	1572	3951	371
50-75% assembled isoforms	2380	2458	471
75-95% assembled isoforms	4597	4269	617
95-100% assembled isoforms	9173	9143	1030
Mean isoform assembly	0.855	0.775	0.795
0-50% matched transcripts	5156	6578	204
50-75% matched transcripts	5739	3356	126
75-95% matched transcripts	9454	4814	203
95-100% matched transcripts	24821	15300	2709
Unannotated	594	11959	137
Mean fraction of transcript matched	0.832	0.544	0.883

This table compares the rnaQUAST evaluation results for Scallop-LR, StringTie, and Iso-Seq Analysis on a human dataset. Metrics descriptions are the same as in Table S7.1.

Table S7.5: rnaQUAST evaluation results for human dataset SAMN04169050, comparing Scallop-LR, StringTie, and Iso-Seq Analysis

Metrics	Scallop-LR	StringTie	Iso-Seq
# Transcripts	38737	33225	15752
Aligned	38734	33217	15752
Uniquely aligned	38727	33099	15750
Unaligned	3	8	0
Misassemblies	6	7	0
0-50% assembled isoforms	1203	3458	1387
50-75% assembled isoforms	1691	1917	1596
75-95% assembled isoforms	3377	3015	2686
95-100% assembled isoforms	9238	8800	4378
Mean isoform assembly	0.877	0.784	0.809
0-50% matched transcripts	6034	6593	580
50-75% matched transcripts	5428	3086	506
75-95% matched transcripts	8205	4216	1232
95-100% matched transcripts	18839	11650	13320
Unannotated	221	7662	114
Mean fraction of transcript matched	0.802	0.558	0.939

This table compares the rnaQUAST evaluation results for Scallop-LR, StringTie, and Iso-Seq Analysis on a human dataset. Metrics descriptions are the same as in Table S7.1.

Table S7.6: rnaQUAST evaluation results for human dataset SAMN04251426.1, comparing Scallop-LR, StringTie, and Iso-Seq Analysis

Metrics	Scallop-LR	StringTie	Iso-Seq
# Transcripts	33883	41154	15342
Aligned	33882	41147	15342
Uniquely aligned	33881	40883	15322
Unaligned	1	7	0
Misassemblies	1	19	10
0-50% assembled isoforms	1972	5067	1999
50-75% assembled isoforms	2334	2666	1812
75-95% assembled isoforms	3649	3429	2323
95-100% assembled isoforms	6903	7347	3350
Mean isoform assembly	0.815	0.714	0.747
0-50% matched transcripts	4722	7576	1554
50-75% matched transcripts	4542	3059	865
75-95% matched transcripts	6157	3734	1385
95-100% matched transcripts	17879	11661	10297
Unannotated	581	15084	1228
Mean fraction of transcript matched	0.807	0.442	0.806

This table compares the rnaQUAST evaluation results for Scallop-LR, StringTie, and Iso-Seq Analysis on a human dataset. Metrics descriptions are the same as in Table S7.1.

Table S7.7: rnaQUAST evaluation results for human dataset SAMN04251426.2, comparing Scallop-LR, StringTie, and Iso-Seq Analysis

Metrics	Scallop-LR	StringTie	Iso-Seq
# Transcripts	33588	41008	16119
Aligned	33587	41004	16119
Uniquely aligned	33578	40814	16104
Unaligned	1	4	0
Misassemblies	6	11	7
0-50% assembled isoforms	1903	5087	2051
50-75% assembled isoforms	2201	2609	1827
75-95% assembled isoforms	3602	3276	2418
95-100% assembled isoforms	7051	7377	3477
Mean isoform assembly	0.821	0.713	0.748
0-50% matched transcripts	4834	7801	1615
50-75% matched transcripts	4525	3048	925
75-95% matched transcripts	6174	3653	1393
95-100% matched transcripts	17429	11337	10787
Unannotated	616	15143	1389
Mean fraction of transcript matched	0.802	0.435	0.8

This table compares the rnaQUAST evaluation results for Scallop-LR, StringTie, and Iso-Seq Analysis on a human dataset. Metrics descriptions are the same as in Table S7.1.

Table S7.8: rnaQUAST evaluation results for human dataset SAMN04251426.3, comparing Scallop-LR, StringTie, and Iso-Seq Analysis

Metrics	Scallop-LR	StringTie	Iso-Seq
# Transcripts	33699	41038	16328
Aligned	33698	41036	16328
Uniquely aligned	33691	40815	16306
Unaligned	1	2	0
Misassemblies	6	16	4
0-50% assembled isoforms	1894	5007	2146
50-75% assembled isoforms	2236	2616	1834
75-95% assembled isoforms	3486	3416	2417
95-100% assembled isoforms	7089	7384	3471
Mean isoform assembly	0.821	0.717	0.744
0-50% matched transcripts	4595	7693	1707
50-75% matched transcripts	4538	3008	955
75-95% matched transcripts	6329	3757	1469
95-100% matched transcripts	17653	11587	10747
Unannotated	576	14965	1441
Mean fraction of transcript matched	0.808	0.441	0.794

This table compares the rnaQUAST evaluation results for Scallop-LR, StringTie, and Iso-Seq Analysis on a human dataset. Metrics descriptions are the same as in Table S7.1.

Table S7.9: rnaQUAST evaluation results for human dataset SAMN04251426.4, comparing Scallop-LR, StringTie, and Iso-Seq Analysis

Metrics	Scallop-LR	StringTie	Iso-Seq
# Transcripts	32952	40754	16179
Aligned	32951	40749	16179
Uniquely aligned	32943	40528	16157
Unaligned	1	5	0
Misassemblies	7	11	7
0-50% assembled isoforms	1940	5087	2109
50-75% assembled isoforms	2246	2729	1852
75-95% assembled isoforms	3563	3361	2430
95-100% assembled isoforms	6970	7220	3414
Mean isoform assembly	0.818	0.711	0.743
0-50% matched transcripts	4634	7569	1647
50-75% matched transcripts	4322	3079	910
75-95% matched transcripts	6116	3565	1440
95-100% matched transcripts	17260	11484	10837
Unannotated	611	15037	1334
Mean fraction of transcript matched	0.806	0.438	0.803

This table compares the rnaQUAST evaluation results for Scallop-LR, StringTie, and Iso-Seq Analysis on a human dataset. Metrics descriptions are the same as in Table S7.1.

Table S7.10: rnaQUAST evaluation results for human dataset SAMN00001694, comparing Scallop-LR, StringTie, and Iso-Seq Analysis

Metrics	Scallop-LR	StringTie	Iso-Seq
# Transcripts	24956	81557	10813
Aligned	24956	81554	10813
Uniquely aligned	24948	81117	10802
Unaligned	0	3	0
Misassemblies	7	9	0
0-50% assembled isoforms	1547	8224	1070
50-75% assembled isoforms	2016	3162	1263
75-95% assembled isoforms	2967	3329	2025
95-100% assembled isoforms	6470	7697	3107
Mean isoform assembly	0.83	0.64	0.802
0-50% matched transcripts	2015	11463	466
50-75% matched transcripts	2582	2822	398
75-95% matched transcripts	5034	3726	762
95-100% matched transcripts	15137	11144	8916
Unannotated	180	52379	270
Mean fraction of transcript matched	0.868	0.219	0.915

This table compares the rnaQUAST evaluation results for Scallop-LR, StringTie, and Iso-Seq Analysis on a human dataset. Metrics descriptions are the same as in Table S7.1.

Table S7.11: rnaQUAST evaluation results for human dataset SAMN00001695, comparing Scallop-LR, StringTie, and Iso-Seq Analysis

Metrics	Scallop-LR	StringTie	Iso-Seq
# Transcripts	21741	107273	12057
Aligned	21741	107269	12057
Uniquely aligned	21736	106575	12039
Unaligned	0	4	0
Misassemblies	3	15	1
0-50% assembled isoforms	1469	10422	1269
50-75% assembled isoforms	1808	3535	1356
75-95% assembled isoforms	2723	3400	2179
95-100% assembled isoforms	6144	8079	3138
Mean isoform assembly	0.832	0.607	0.786
0-50% matched transcripts	1498	15153	606
50-75% matched transcripts	2146	2924	367
75-95% matched transcripts	4350	3529	829
95-100% matched transcripts	13582	10679	9484
Unannotated	161	74942	770
Mean fraction of transcript matched	0.879	0.165	0.872

This table compares the rnaQUAST evaluation results for Scallop-LR, StringTie, and Iso-Seq Analysis on a human dataset. Metrics descriptions are the same as in Table S7.1.

Table S7.12: rnaQUAST evaluation results for human dataset SAMN00001696, comparing Scallop-LR, StringTie, and Iso-Seq Analysis

Metrics	Scallop-LR	StringTie	Iso-Seq
# Transcripts	16487	65425	8855
Aligned	16487	65418	8855
Uniquely aligned	16482	65094	8847
Unaligned	0	7	0
Misassemblies	3	4	1
0-50% assembled isoforms	1183	7195	859
50-75% assembled isoforms	1482	2726	1004
75-95% assembled isoforms	2364	2904	1749
95-100% assembled isoforms	5115	6335	2604
Mean isoform assembly	0.834	0.633	0.807
0-50% matched transcripts	1066	9110	350
50-75% matched transcripts	1454	2074	240
75-95% matched transcripts	3011	2636	573
95-100% matched transcripts	10875	9927	7369
Unannotated	77	41656	321
Mean fraction of transcript matched	0.891	0.227	0.911

This table compares the rnaQUAST evaluation results for Scallop-LR, StringTie, and Iso-Seq Analysis on a human dataset. Metrics descriptions are the same as in Table S7.1.

Table S7.13: rnaQUAST evaluation results for human dataset SAMN00006465, comparing Scallop-LR, StringTie, and Iso-Seq Analysis

Metrics	Scallop-LR	StringTie	Iso-Seq
# Transcripts	20038	82621	12557
Aligned	20038	82617	12557
Uniquely aligned	20033	82068	12546
Unaligned	0	4	0
Misassemblies	4	11	1
0-50% assembled isoforms	1333	8262	1180
50-75% assembled isoforms	1630	2921	1348
75-95% assembled isoforms	2517	3083	2176
95-100% assembled isoforms	6315	7746	3520
Mean isoform assembly	0.842	0.636	0.804
0-50% matched transcripts	1375	11130	426
50-75% matched transcripts	1856	2375	397
75-95% matched transcripts	3993	3258	854
95-100% matched transcripts	12688	11297	10512
Unannotated	122	54521	367
Mean fraction of transcript matched	0.882	0.207	0.921

This table compares the rnaQUAST evaluation results for Scallop-LR, StringTie, and Iso-Seq Analysis on a human dataset. Metrics descriptions are the same as in Table S7.1.

Table S7.14: rnaQUAST evaluation results for human dataset SAMN00006466, comparing Scallop-LR, StringTie, and Iso-Seq Analysis

Metrics	Scallop-LR	StringTie	Iso-Seq
# Transcripts	18192	140280	12335
Aligned	18190	140274	12335
Uniquely aligned	18187	139647	12307
Unaligned	2	6	0
Misassemblies	1	18	1
0-50% assembled isoforms	1315	13372	1414
50-75% assembled isoforms	1502	3942	1229
75-95% assembled isoforms	2481	3485	2072
95-100% assembled isoforms	5899	8561	3280
Mean isoform assembly	0.84	0.574	0.782
0-50% matched transcripts	1300	20842	701
50-75% matched transcripts	1649	3109	391
75-95% matched transcripts	3547	3061	749
95-100% matched transcripts	11541	9780	9300
Unannotated	151	103450	1193
Mean fraction of transcript matched	0.879	0.122	0.833

This table compares the rnaQUAST evaluation results for Scallop-LR, StringTie, and Iso-Seq Analysis on a human dataset. Metrics descriptions are the same as in Table S7.1.

Table S7.15: rnaQUAST evaluation results for human dataset SAMN00006467, comparing Scallop-LR, StringTie, and Iso-Seq Analysis

Metrics	Scallop-LR	StringTie	Iso-Seq
# Transcripts	15914	134501	10487
Aligned	15914	134492	10487
Uniquely aligned	15912	133856	10458
Unaligned	0	9	0
Misassemblies	2	17	0
0-50% assembled isoforms	1199	13214	1160
50-75% assembled isoforms	1396	3910	1081
75-95% assembled isoforms	2322	3441	1846
95-100% assembled isoforms	5296	8069	2925
Mean isoform assembly	0.839	0.567	0.79
0-50% matched transcripts	1202	20694	674
50-75% matched transcripts	1463	2767	281
75-95% matched transcripts	2848	2744	574
95-100% matched transcripts	10264	8927	7765
Unannotated	135	99314	1192
Mean fraction of transcript matched	0.878	0.117	0.813

This table compares the rnaQUAST evaluation results for Scallop-LR, StringTie, and Iso-Seq Analysis on a human dataset. Metrics descriptions are the same as in Table S7.1.

Table S7.16: rnaQUAST evaluation results for human dataset SAMN00006579, comparing Scallop-LR, StringTie, and Iso-Seq Analysis

Metrics	Scallop-LR	StringTie	Iso-Seq
# Transcripts	20769	124624	13147
Aligned	20769	124621	13147
Uniquely aligned	20768	123959	13128
Unaligned	0	3	0
Misassemblies	1	25	4
0-50% assembled isoforms	1214	12066	1231
50-75% assembled isoforms	1516	3755	1299
75-95% assembled isoforms	2494	3507	2106
95-100% assembled isoforms	6387	8960	3508
Mean isoform assembly	0.85	0.597	0.798
0-50% matched transcripts	1882	20058	848
50-75% matched transcripts	2384	2921	545
75-95% matched transcripts	4223	3167	895
95-100% matched transcripts	12105	9394	9655
Unannotated	174	89030	1199
Mean fraction of transcript matched	0.855	0.133	0.829

This table compares the rnaQUAST evaluation results for Scallop-LR, StringTie, and Iso-Seq Analysis on a human dataset. Metrics descriptions are the same as in Table S7.1.

Table S7.17: rnaQUAST evaluation results for human dataset SAMN00006580, comparing Scallop-LR, StringTie, and Iso-Seq Analysis

Metrics	Scallop-LR	StringTie	Iso-Seq
# Transcripts	18480	152741	12817
Aligned	18480	152739	12817
Uniquely aligned	18477	152257	12795
Unaligned	0	2	0
Misassemblies	1	17	5
0-50% assembled isoforms	1246	14212	1290
50-75% assembled isoforms	1497	4284	1192
75-95% assembled isoforms	2438	3834	2001
95-100% assembled isoforms	5809	9259	3276
Mean isoform assembly	0.842	0.578	0.788
0-50% matched transcripts	1688	24592	1023
50-75% matched transcripts	1898	3141	427
75-95% matched transcripts	3507	2930	739
95-100% matched transcripts	11154	8948	8562
Unannotated	230	113096	2059
Mean fraction of transcript matched	0.856	0.108	0.75

This table compares the rnaQUAST evaluation results for Scallop-LR, StringTie, and Iso-Seq Analysis on a human dataset. Metrics descriptions are the same as in Table S7.1.

Table S7.18: rnaQUAST evaluation results for human dataset SAMN00006581, comparing Scallop-LR, StringTie, and Iso-Seq Analysis

Metrics	Scallop-LR	StringTie	Iso-Seq
# Transcripts	20476	125563	12512
Aligned	20476	125556	12512
Uniquely aligned	20475	125049	12495
Unaligned	0	7	0
Misassemblies	1	17	2
0-50% assembled isoforms	1279	11940	1195
50-75% assembled isoforms	1582	3652	1243
75-95% assembled isoforms	2452	3340	2128
95-100% assembled isoforms	6273	8602	3409
Mean isoform assembly	0.844	0.592	0.799
0-50% matched transcripts	2036	19464	878
50-75% matched transcripts	2213	2901	417
75-95% matched transcripts	4104	2967	812
95-100% matched transcripts	11946	9341	9311
Unannotated	176	90846	1091
Mean fraction of transcript matched	0.852	0.13	0.832

This table compares the rnaQUAST evaluation results for Scallop-LR, StringTie, and Iso-Seq Analysis on a human dataset. Metrics descriptions are the same as in Table S7.1.

Table S8.1: rnaQUAST evaluation results for mouse dataset SAMEA3374575, comparing Scallop-LR, StringTie, and Iso-Seq Analysis

Metrics	Scallop-LR	StringTie	Iso-Seq
# Transcripts	6890	12610	4017
Aligned	6890	12610	4017
Uniquely aligned	6886	12528	4013
Unaligned	0	0	0
Misassemblies	4	1	0
0-50% assembled isoforms	703	2605	606
50-75% assembled isoforms	761	1258	520
75-95% assembled isoforms	1194	1597	1003
95-100% assembled isoforms	2451	4101	1248
Mean isoform assembly	0.818	0.729	0.779
0-50% matched transcripts	243	765	41
50-75% matched transcripts	369	899	155
75-95% matched transcripts	920	1738	294
95-100% matched transcripts	5323	8108	3510
Unannotated	31	1098	16
Mean fraction of transcript matched	0.931	0.819	0.96

This table compares the rnaQUAST evaluation results for Scallop-LR, StringTie, and Iso-Seq Analysis on a mouse dataset. Metrics descriptions are the same as in Table S7.1.

Table S8.2: rnaQUAST evaluation results for mouse dataset SAMEA3374576, comparing Scallop-LR, StringTie, and Iso-Seq Analysis

Metrics	Scallop-LR	StringTie	Iso-Seq
# Transcripts	6720	12207	4164
Aligned	6720	12206	4164
Uniquely aligned	6715	12104	4163
Unaligned	0	1	0
Misassemblies	5	0	1
0-50% assembled isoforms	699	2406	616
50-75% assembled isoforms	706	1245	585
75-95% assembled isoforms	1155	1632	975
95-100% assembled isoforms	2563	4194	1385
Mean isoform assembly	0.825	0.743	0.783
0-50% matched transcripts	176	700	48
50-75% matched transcripts	275	815	71
75-95% matched transcripts	899	1657	279
95-100% matched transcripts	5334	7967	3749
Unannotated	31	1067	16
Mean fraction of transcript matched	0.94	0.817	0.969

This table compares the rnaQUAST evaluation results for Scallop-LR, StringTie, and Iso-Seq Analysis on a mouse dataset. Metrics descriptions are the same as in Table S7.1.

Table S8.3: rnaQUAST evaluation results for mouse dataset SAMEA3374577, comparing Scallop-LR, StringTie, and Iso-Seq Analysis

Metrics	Scallop-LR	StringTie	Iso-Seq
# Transcripts	5972	11247	3699
Aligned	5972	11247	3699
Uniquely aligned	5967	11193	3695
Unaligned	0	0	0
Misassemblies	4	1	1
0-50% assembled isoforms	653	2250	554
50-75% assembled isoforms	662	1169	505
75-95% assembled isoforms	1078	1531	908
95-100% assembled isoforms	2343	3931	1222
Mean isoform assembly	0.823	0.744	0.784
0-50% matched transcripts	180	667	60
50-75% matched transcripts	236	765	83
75-95% matched transcripts	732	1454	230
95-100% matched transcripts	4796	7442	3305
Unannotated	23	918	20
Mean fraction of transcript matched	0.942	0.826	0.963

This table compares the rnaQUAST evaluation results for Scallop-LR, StringTie, and Iso-Seq Analysis on a mouse dataset. Metrics descriptions are the same as in Table S7.1.

Table S8.4: rnaQUAST evaluation results for mouse dataset SAMEA3374578, comparing Scallop-LR, StringTie, and Iso-Seq Analysis

Metrics	Scallop-LR	StringTie	Iso-Seq
# Transcripts	5783	10979	3492
Aligned	5783	10979	3492
Uniquely aligned	5778	10925	3491
Unaligned	0	0	0
Misassemblies	5	0	0
0-50% assembled isoforms	625	2378	543
50-75% assembled isoforms	604	1166	466
75-95% assembled isoforms	989	1458	860
95-100% assembled isoforms	2169	3674	1154
Mean isoform assembly	0.82	0.726	0.782
0-50% matched transcripts	160	696	23
50-75% matched transcripts	239	727	57
75-95% matched transcripts	842	1550	210
95-100% matched transcripts	4503	7184	3184
Unannotated	34	822	18
Mean fraction of transcript matched	0.936	0.83	0.973

This table compares the rnaQUAST evaluation results for Scallop-LR, StringTie, and Iso-Seq Analysis on a mouse dataset. Metrics descriptions are the same as in Table S7.1.

Table S8.5: rnaQUAST evaluation results for mouse dataset SAMEA3374579, comparing Scallop-LR, StringTie, and Iso-Seq Analysis

Metrics	Scallop-LR	StringTie	Iso-Seq
# Transcripts	8052	16520	4609
Aligned	8052	16520	4609
Uniquely aligned	8052	16414	4600
Unaligned	0	0	0
Misassemblies	0	0	0
0-50% assembled isoforms	949	3648	835
50-75% assembled isoforms	1006	1687	650
75-95% assembled isoforms	1433	1994	1111
95-100% assembled isoforms	3104	5197	1502
Mean isoform assembly	0.812	0.714	0.763
0-50% matched transcripts	155	1046	26
50-75% matched transcripts	288	1119	82
75-95% matched transcripts	989	2223	268
95-100% matched transcripts	6598	10605	4213
Unannotated	22	1526	19
Mean fraction of transcript matched	0.952	0.813	0.972

This table compares the rnaQUAST evaluation results for Scallop-LR, StringTie, and Iso-Seq Analysis on a mouse dataset. Metrics descriptions are the same as in Table S7.1.

Table S8.6: rnaQUAST evaluation results for mouse dataset SAMEA3374580, comparing Scallop-LR, StringTie, and Iso-Seq Analysis

Metrics	Scallop-LR	StringTie	Iso-Seq
# Transcripts	8206	18482	4679
Aligned	8206	18480	4679
Uniquely aligned	8206	18301	4670
Unaligned	0	2	0
Misassemblies	0	0	1
0-50% assembled isoforms	1057	4217	1028
50-75% assembled isoforms	1016	1811	627
75-95% assembled isoforms	1491	1961	1047
95-100% assembled isoforms	3058	5173	1408
Mean isoform assembly	0.802	0.692	0.729
0-50% matched transcripts	149	1199	42
50-75% matched transcripts	281	1196	57
75-95% matched transcripts	960	2229	257
95-100% matched transcripts	6794	11398	4297
Unannotated	22	2457	22
Mean fraction of transcript matched	0.955	0.771	0.972

This table compares the rnaQUAST evaluation results for Scallop-LR, StringTie, and Iso-Seq Analysis on a mouse dataset. Metrics descriptions are the same as in Table S7.1.

Table S8.7: rnaQUAST evaluation results for mouse dataset SAMEA3374581, comparing Scallop-LR, StringTie, and Iso-Seq Analysis

Metrics	Scallop-LR	StringTie	Iso-Seq
# Transcripts	9851	20349	5765
Aligned	9851	20348	5765
Uniquely aligned	9848	20157	5759
Unaligned	0	1	0
Misassemblies	2	2	0
0-50% assembled isoforms	1199	4489	1252
50-75% assembled isoforms	1137	1932	743
75-95% assembled isoforms	1579	2038	1204
95-100% assembled isoforms	3680	5704	1695
Mean isoform assembly	0.808	0.696	0.728
0-50% matched transcripts	221	1312	57
50-75% matched transcripts	418	1347	108
75-95% matched transcripts	1373	2690	384
95-100% matched transcripts	7792	12367	5166
Unannotated	44	2629	50
Mean fraction of transcript matched	0.944	0.772	0.964

This table compares the rnaQUAST evaluation results for Scallop-LR, StringTie, and Iso-Seq Analysis on a mouse dataset. Metrics descriptions are the same as in Table S7.1.

Table S8.8: rnaQUAST evaluation results for mouse dataset SAMEA3374582, comparing Scallop-LR, StringTie, and Iso-Seq Analysis

Metrics	Scallop-LR	StringTie	Iso-Seq
# Transcripts	8071	18712	4748
Aligned	8071	18712	4748
Uniquely aligned	8069	18543	4740
Unaligned	0	0	0
Misassemblies	1	1	0
0-50% assembled isoforms	1115	4837	1344
50-75% assembled isoforms	1081	1980	619
75-95% assembled isoforms	1335	1892	919
95-100% assembled isoforms	2718	4581	1187
Mean isoform assembly	0.784	0.657	0.674
0-50% matched transcripts	98	1091	36
50-75% matched transcripts	243	1166	43
75-95% matched transcripts	905	2275	170
95-100% matched transcripts	6806	11737	4444
Unannotated	17	2439	55
Mean fraction of transcript matched	0.962	0.779	0.97

This table compares the rnaQUAST evaluation results for Scallop-LR, StringTie, and Iso-Seq Analysis on a mouse dataset. Metrics descriptions are the same as in Table S7.1.

Table S9: SRA information for the 26 datasets used in this study

Dataset	BioSample	SRA Study	Organism	Year	Instrument
1	SAMN00001694	ERP015321	*Homo sapiens*	2016	RS
2	SAMN00001695	ERP015321	*Homo sapiens*	2016	RS
3	SAMN00001696	ERP015321	*Homo sapiens*	2016	RS
4	SAMN00006465	ERP015321	*Homo sapiens*	2016	RS
5	SAMN00006466	ERP015321	*Homo sapiens*	2016	RS
6	SAMN00006467	ERP015321	*Homo sapiens*	2016	RS
7	SAMN00006579	ERP015321	*Homo sapiens*	2016	RS
8	SAMN00006580	ERP015321	*Homo sapiens*	2016	RS
9	SAMN00006581	ERP015321	*Homo sapiens*	2016	RS
10	SAMN08182059	SRP126849	*Homo sapiens*	2017	RS II
11	SAMN08182060	SRP126849	*Homo sapiens*	2017	RS II
12	SAMN04563763	SRP071928	*Homo sapiens*	2016	RS II
13	SAMN07611993	SRP098984	*Homo sapiens*	2018	RS II
14	SAMN04169050	SRP068953	*Homo sapiens*	2016	RS II
15	SAMN04251426.1	SRP065930	*Homo sapiens*	2016	RS II
16	SAMN04251426.2	SRP065930	*Homo sapiens*	2016	RS II
17	SAMN04251426.3	SRP065930	*Homo sapiens*	2016	RS II
18	SAMN04251426.4	SRP065930	*Homo sapiens*	2016	RS II
19	SAMEA3374575	ERP010189	*Mus musculus*	2015	RS
20	SAMEA3374576	ERP010189	*Mus musculus*	2015	RS
21	SAMEA3374577	ERP010189	*Mus musculus*	2015	RS
22	SAMEA3374578	ERP010189	*Mus musculus*	2015	RS
23	SAMEA3374579	ERP010189	*Mus musculus*	2015	RS
24	SAMEA3374580	ERP010189	*Mus musculus*	2015	RS
25	SAMEA3374581	ERP010189	*Mus musculus*	2015	RS
26	SAMEA3374582	ERP010189	*Mus musculus*	2015	RS

This table summarizes the 26 datasets used in this paper. 18 datasets are human and eight datasets are mouse. The data were downloaded from the corresponding SRA Study. The multiple SRA Runs for each BioSample under the corresponding SRA Study were extracted, processed, and then merged into a large dataset.

Table S10: Performance comparison of Scallop-LR vs. Scallop on human data

Datasets	Sensitivity (%)		Precision (%)		# Total Multi-Exon Transcripts		# Correctly Predicted Known Transcripts	
	Scallop-LR	Scallop	Scallop-LR	Scallop	Scallop-LR	Scallop	Scallop-LR	Scallop
SAMN00001694	5.50	5.38	38.47	34.57	24903	27070	9580	9357
SAMN00001695	5.36	5.29	42.96	36.82	21731	25013	9336	9211
SAMN00001696	4.48	4.48	47.29	41.01	16476	19030	7791	7805
SAMN00006465	5.32	5.36	46.28	40.69	20019	22950	9264	9339
SAMN00006466	5.05	5.05	48.42	38.91	18171	22608	8798	8797
SAMN00006467	4.62	4.58	50.57	39.95	15900	19953	8040	7972
SAMN00006579	5.19	5.13	43.52	35.87	20742	24908	9027	8934
SAMN00006580	4.87	4.81	45.89	35.43	18467	23638	8474	8376
SAMN00006581	5.09	5.07	43.31	35.03	20458	25194	8861	8826
SAMN08182059	5.29	5.13	36.34	31.37	25332	28454	9206	8927
SAMN08182060	5.52	5.41	43.59	36.01	22038	26129	9606	9410
SAMN04563763	4.87	4.92	46.90	41.09	18078	20850	8478	8567
SAMN07611993	7.60	7.53	28.97	27.02	45657	48531	13226	13113
SAMN04169050	6.86	6.63	30.90	25.72	38657	44848	11946	11536
SAMN04251426.1	5.70	5.57	29.32	28.25	33824	34343	9916	9701
SAMN04251426.2	5.76	5.66	29.92	28.83	33534	34155	10034	9846
SAMN04251426.3	5.81	5.75	30.09	29.42	33637	33995	10122	10001
SAMN04251426.4	5.78	5.67	30.59	29.32	32908	33647	10065	9864

The above table compares the performance of Scallop-LR (v0.9.1) with the performance of Scallop (v0.10.3) using the Gffcompare evaluation. The same 18 human PacBio datasets as described in Table S1 were evaluated. The parameter settings (options) used for Scallop (v0.10.3) are “--max_num_cigar 1000” and “--min_num_hits_in_bundle 1”.

Table S11: Comparison of Scallop-LR with clustering vs. Scallop-LR without clustering on human data

Datasets	# Total Multi-Exon Transcripts		# Correctly Predicted Known Transcripts		% of Correctly Assembled Known Transcripts Missing Due to Clustering	% of Nearly Redundant Transcripts Removed by Clustering
	Scallop-LR with Clustering	Scallop-LR without Clustering	Scallop-LR with Clustering	Scallop-LR without Clustering		
SAMN00001694	24903	26853	9580	9770	1.94	10.3
SAMN00001695	21731	23508	9336	9479	1.51	11.65
SAMN00001696	16476	17695	7791	7937	1.84	11.0
SAMN00006465	20019	21504	9264	9452	1.99	10.76
SAMN00006466	18171	19513	8798	8955	1.75	11.22
SAMN00006467	15900	17007	8040	8157	1.43	11.19
SAMN00006579	20742	22229	9027	9201	1.89	10.08
SAMN00006580	18467	19745	8474	8622	1.72	10.16
SAMN00006581	20458	21994	8861	9033	1.9	10.52
SAMN08182059	25332	27415	9206	9340	1.43	10.78
SAMN08182060	22038	24449	9606	9793	1.91	15.17
SAMN04563763	18078	19493	8478	8634	1.81	11.59
SAMN07611993	45657	50082	13226	13549	2.38	11.23
SAMN04169050	38657	43806	11946	12188	1.99	15.52
SAMN04251426.1	33824	36534	9916	10083	1.66	9.61
SAMN04251426.2	33534	36225	10034	10234	1.95	9.58
SAMN04251426.3	33637	36190	10122	10283	1.57	9.23
SAMN04251426.4	32908	35450	10065	10287	2.16	9.22

The above table compares the results of Scallop-LR without post-assembly clustering with the results of Scallop-LR with post-assembly clustering (using the default "--max_cluster_intron_distance") by using the Gffcompare evaluation. The same 18 human PacBio datasets as described in Table S1 were evaluated. The percentages are computed as the following:

"% of Correctly Assembled Known Transcripts Missing Due to Clustering" = 100 × (("# matching transcripts without clustering" – "# matching transcripts with clustering") / "# matching transcripts without clustering").

"% of Nearly Redundant Transcripts Removed by Clustering" = 100 × (("# non-matching transcripts without clustering" – "# non-matching transcripts with clustering") / "# non-matching transcripts without clustering").

Where "# non-matching transcripts" = "# Total Multi-Exon Transcripts" – "# matching transcripts";
"# matching transcripts" = "# Correctly Predicted Known Transcripts".

Table S12: Simulated Human Data: Sensitivity, Precision, Correctly Predicted Known Transcripts, and Total Multi-Exon Transcripts of Scallop-LR and StringTie

Dataset	Sensitivity (%)		Precision (%)		# Total Multi-Exon Transcripts		# Correctly Predicted Known Transcripts	
	Scallop-LR	StringTie	Scallop-LR	StringTie	Scallop-LR	StringTie	Scallop-LR	StringTie
Simulated Human CCS Reads	53.05	45.03	66.54	63.51	6226	5538	4143	3517

This table compares the Gffcompare evaluation results for Scallop-LR and StringTie on a simulated human dataset (Liu *et al.*, 2019). The transcriptome that was used to generate the simulated long reads originated from the Ensembl annotation *Homo sapiens* GRCh38.94 and was a subset of the transcripts in this Ensembl annotation, by removing unfinished scaffolds, transcripts shorter than 200 bp, annotations with an unknown reference, etc. and randomly selecting alternative-splicing genes, single-splicing genes, and genes with small exons (< 31bp). The PacBio PBSIM tool was used to generate the simulated CCS reads from this transcriptome. The simulation was model-based using the CCS model, and three runs of simulations were performed by using three different sequencing depths 4X, 10X and 30X respectively. We merged the CCS reads generated with the three sequencing depths together to obtain this simulated human dataset. We used the transcripts in the transcriptome sequences that were used to generate the simulated CCS reads to extract the transcripts' records and their corresponding genes' records from the Ensembl annotation *Homo sapiens* GRCh38.94 to obtain an annotation GTF file. This extracted annotation GTF file serves as the reference in Gffcompare and the "ground truth", and it contains 7810 multi-exon transcripts.

Table S13: rnaQUAST evaluation results for a simulated human dataset, comparing Scallop-LR and StringTie

Metrics	Scallop-LR	StringTie
# Transcripts	6228	7246
Aligned	6228	7246
Uniquely aligned	6225	7120
Unaligned	0	0
Misassemblies	0	0
0-50% assembled isoforms	90	41
50-75% assembled isoforms	171	48
75-95% assembled isoforms	253	170
95-100% assembled isoforms	3103	4248
Mean isoform assembly	0.956	0.984
0-50% matched transcripts	107	41
50-75% matched transcripts	418	217
75-95% matched transcripts	1668	1039
95-100% matched transcripts	4027	4979
Unannotated	5	967
Mean fraction of transcript matched	0.924	0.81

This table compares the rnaQUAST evaluation results for Scallop-LR and StringTie on a simulated human dataset. The same simulated human dataset as described in Table S12 was evaluated. The same extracted annotation GTF file as described in Table S12 was used to generate the gene annotation database, which was used by rnaQUAST. Metrics descriptions are the same as in Table S7.1.
