# Supplementary material for: Quantifying the benefit offered by transcript assembly with Scallop-LR on single-molecule long reads
Source: Genome Biol. 2019 Dec 18;20:287. doi: 10.1186/s13059-019-1883-0 (PMC6918626; doi:10.1186/s13059-019-1883-0)
Supplement: Supplementary file 2 — Additional file 2 Review history [file 13059_2019_1883_MOESM2_ESM.docx]

**1^st^ round**

**Reviewer 1**

In this paper, the authors proposed a new assembler, Scallop-LR, by employing the Scallop procedure developed by the same group on a set of single-molecule long reads to improve on the sensitivity of assembling transcriptome. The topic is still interesting even though the third generation sequencing data has been generated in a large scale. As far as I know, this is the first tool tailed to assemble long sequencing reads. It does outperform previous ones in assembling long sequencing reads, but I have some concerns about the algorithm design.

1. Line 33 Page 7, the authors mentioned that Scallop-LR deals with the high error rates in long read. However, I did not find the details of dealing with errors. As is known, CCS reads also contains a lot of sequencing errors, please describe the details of how to handle the errors.

2. Line 21 Page 7, about the very similar splice positions, the authors cluster them into a single transcript. I think this is dangerous for reference-based assembly since there are many real transcripts with very similar splice positions. So please demonstrate the percentage of correctly assembled transcripts that are missing because of the clustering by using Gffcompare? And similarly the percentage of wrongly assembled (or nearly redundant) transcripts that are removed due to the clustering.

3. How to cluster the very similar transcripts in detail? As their splice positions are different, which one do you choose for the clustered consensus single transcript?

4. Line 46 Page 6, how to understand the phrase "within a region"? And also, in the next paragraph, what do the x1, x2, x3 mean? And what is [a,c]? Please describe this paragraph in detail.

5. The authors used Minimap2 to align CCS reads without error correction. How is the mapping rate? If the error-corrected CCS were used for mapping, whether or not the assembly accuracy would be increased?

6. Line 16 Page 5, the authors say that "vertices are exons". As I know, each node in the graph is a continuous region in the reference genome. It is hard to say that each constructed vertex corresponds a real exon.

7. I have a question that if there are some CCS reads mapped to multiple positions in the reference genome? If so, how do you deal with it?

**Reviewer 2**

The paper entitled “Quantifying the Benefit Offered by Transcript Assembly on Single-Molecule Long Reads” describes a novel algorithm for reference-based assembly of long error-prone RNA reads. The authors extend their previous short-read assembler Scallop by adding several modification, that allow to benefit from reads covering several splice junctions and “boundary reads” containing 5’ or/and 3’ primers. Since protocols for sequencing long RNA reads appeared rather recently, very few methods for its processing exist at the moment, so a high-quality long-read transcriptome assembler is a valuable instrument for the scientific community.

The authors use different existing methods for quality evaluation on multiple datasets against the best available tools for assembling long RNA reads. Presented benchmarks seem to be fair and strongly support Scallop-LR good performance in comparison to other tools. What I find important, is that the authors upload all scripts and instructions necessary for experiment reproduction, something that should become a standard for all “tool papers”. However, I encountered some issues while running the analysis (see comments below). The tool itself is available, documented and working, which, indeed, adds value to this work. Some dependencies (like Iso-seq pipeline) are a bit heavy, but looks like there is no other choice so far.

Overall, the paper is well-structured and relatively easy to follow. Below I summarize a few non-crucial issues and questions, which, in my opinion, may improve the quality of the current Manuscript.

Major comments

● Since the novel method is entirely based on the previous tool Scallop, the manuscript includes the description only for the key modifications introduced in Scallop-LR. Indeed, understanding of some novel features would require complete description of the previous algorithm. For example, it is not entirely clear how exactly phasing paths are preserved by the graph decomposition algorithm. Presenting the entire previous algorithm in detail is probably unnecessary, but I feel like section 2.1 would significantly benefit from a figure showing some simple example of a splice graph and its decomposition without and with long reads. Some algorithmic details in the supplementary material may also be an option.

● Although it seems intuitive that Scallop-LR should outperform original Scallop, explicitly showing quality increase could be a significant justification for added algorithms.

● As one of the quality metrics, the authors use the number of x-y% matched transcripts. However, this metric is not normalized anyhow. For example, an assembler that simply duplicates every assembled transcript would have twice more matched transcripts. Worth noting, that Scallop-LR typically has fewer transcripts in its assembly than StringTie, but still outperforms it by the number of x-y% matched transcripts. Thus, normalization of this metric would not change the claim that Scallop-LR outperforms StringTie according to this parameter.

● As stated in the paper, according to a very important metric “x-y% assembled isoforms” StringTie typically has a better assembly than Scallop-LR. It would be useful to check whether StringTie really assembles more isoforms, or it assembles some transcripts that are not in the reads, but were assembled by mistake. To check it, I propose to use simulated data, so that the initial set of expressed isoforms is known (ground truth). Thus, it would be possible to compute the percentage of x-y% assembled isoforms relative to this initial set, rather than all known isoforms. It is also possible to compute the number of isoforms that were falsely assembled (e.g. absent in the simulated data). Based on the statistics presented in the manuscripts, my guess it that StringTie may generate a significant number of falsely assembled isoforms.

By the way, comparison on simulated data can be useful regardless of the comment above.

● Although it took some time, I reproduced the experiments according to instructions here

https://github.com/Kingsford-Group/lrassemblyanalysis . Eventually, I managed to get to the Scallop-LR assembly step. I’d suggest the following:

○ Give more details on how to prepare the reference data (e.g. gmap db and gmap reference sets).

○ Since paths to the reference data are neither provided as parameters nor set in some config file (which would be more convenient), adding details on which lines of code should be changed could ease the process. Looking through the code or error logs can be a bit time consuming.

○ Some binaries are expected to be in $PATH (like minimap2), some have hardcoded local path in the code (Scallop, Gffcompare).

○ Bioawk dependency is not specified.

○ Re-running script biosample_isoseq.sh after a failure gives error messages (tries to create existing directory or links). Not critical, but extra error messages do confuse.

○ Scripts are not compatible with newer smrtlink versions. A note on that can also be added.

Minor comments

● While the term “assembly” relates to two different groups of methods — de novo and reference-based (probably even more often to de novo), I suggest to mention that the developed method is reference-based as early as possible, e.g. in the beginning of the introduction or even in the abstract.

● Section 2.1.2. It is not clear where variables c and d come from. Are those just notations for arbitrary exons starting at position a (ending at position b)?

● Section 2.1.2. Let’s say the allowed variance for transcript start discovery is 5 bp, and we have six 5’ boundary reads aligned to positions [95, x1 ], [98, x2 ], [101, x3 ], [105, x4 ], [107, x5 ], [110, x6 ]. In this case, we can pick, for example, position 105 as a starting position of a transcript, or we can pick two alternative starts 98 and 107 (both supported by 3 reads within variance region). How would algorithm work in this case?

● The authors are honest about lower precision on large datasets for Scallop-LR. It would be interesting to understand the reason of such behaviour. Could it be the effect of using some absolute thresholds in the algorithm?

● It may be useful to mention that an unannotated transcript reported by rnaQUAST denotes an assembled transcripts mapped to intergenic space, and thus does not relate to the number of novel isoforms discovered by other methods. In my opinion, it may possibly be confusing for a reader why the number of novel isoforms is significantly larger than the number of unannotated transcripts, but I leave this decision to the authors

● Although authors mention TransRate, it remains unclear how exactly it was used in quality evaluation.

● GitHub repository does not contain any information on Scallop-LR and its pipeline (except the release archives).

**Response to reviewers**

**Reviewer #1 :** In this paper, the authors proposed a new assembler, Scallop-LR, by employing the Scallop procedure developed by the same group on a set of single-molecule long reads to improve on the sensitivity of assembling transcriptome. The topic is still interesting even though the third generation sequencing data has been generated in a large scale. As far as I know, this is the first tool tailed to assemble long sequencing reads. It does outperform previous ones in assembling long sequencing reads, but I have some concerns about the algorithm design.

Thank you for your positive comments.

1. Line 33 Page 7, the authors mentioned that Scallop-LR deals with the high error rates in long read. However, I did not find the details of dealing with errors. As is known, CCS reads also contains a lot of sequencing errors, please describe the details of how to handle the errors.

Thank you for your suggestion. We now have added more details on how Scallop-LR deals with the high error rates in long reads in the last paragraph of Section 2.1.2. Errors in long reads are mostly insertions and deletions, which are more difficult to correct than substitutions and may lead to mis-alignments around splice positions. More advanced optimizations to correct potentially wrong splice positions from alignments is an interesting direction for future work.

2. Line 21 Page 7, about the very similar splice positions, the authors cluster them into a single transcript. I think this is dangerous for reference-based assembly since there are many real transcripts with very similar splice positions. So please demonstrate the percentage of correctly assembled transcripts that are missing because of the clustering by using Gffcompare? And similarly the percentage of wrongly assembled (or nearly redundant) transcripts that are removed due to the clustering.

Thank you for your suggestion. We have now compared the results of Scallop-LR without the post-assembly clustering with the results of Scallop-LR with the post-assembly clustering, and computed the percentages of correctly assembled known transcripts that are missing because of the clustering and the percentages of wrongly assembled (or nearly redundant) transcripts that are removed due to the clustering for the 18 human datasets. The results are shown in Supplementary Table S11. We discussed these results in the Discussion section. We found that the percentages of correctly assembled known transcripts missing due to clustering are between 1.43% - 2.38% (this percentage < 2% for all datasets except for two), and the percentages of nearly redundant transcripts removed by clustering are between 9.22% - 15.52% (this percentage > 10% for all datasets except for four). These results indicate that the effect of missing correctly assembled known transcripts by the post-assembly clustering is relatively minor, while the post-assembly clustering substantially removes nearly redundant transcripts and significantly improves the precision. Decreasing the allowance for splice positions’ differences could further reduce the effect of missing correctly assembled known transcripts due to clustering, and the users can adjust this parameter for their different interest.

3. How to cluster the very similar transcripts in detail? As their splice positions are different, which one do you choose for the clustered consensus single transcript?

We now have added more detailed explanation on how we cluster the very similar transcripts in the 2nd last paragraph in Section 2.1.2. In short, we use a single-linkage clustering algorithm, and report the transcript with the highest abundance in each cluster as the consensus single transcript for the cluster.

4. Line 46 Page 6, how to understand the phrase "within a region"? And also, in the next paragraph, what do the x1, x2, x3 mean? And what is [a,c]? Please describe this paragraph in detail.

Thank you for your suggestion. We have now made it clearer what the phrase "within a region" means--- we rewrote that sentence as: “Specifically, when there are a certain number of boundary reads whose boundaries align within an exonic region in the genome with very similar boundary positions (the default minimum number is 3), the algorithm defines it as a starting or ending boundary.”

We also added explanations on what the x1, x2, x3 mean and what [a,c] is in this paragraph:

“x1, x2, x3, … are the ending positions of the aligned genomic regions of these reads.”

“we add an edge connecting the source s to the vertex corresponding to the exonic region [a, c] in the genome (where c is the ending position of this exonic region).”

5. The authors used Minimap2 to align CCS reads without error correction. How is the mapping rate? If the error-corrected CCS were used for mapping, whether or not the assembly accuracy would be increased?

The mapping rates of CCS reads by Minimap2 are between 82.31% - 95.98% (excluding the alignments that are secondary or supplementary) for our analyzed 26 datasets (including both human and mouse). All datasets except for only one have the mapping rate > 85%. About 58% of the datasets have the mapping rate > 90%, and about 35% of the datasets have the mapping rate > 95%.

Common error-correction methods use short reads to correct errors in long reads; however, for many of the long-read datasets we analyzed, there are no corresponding short reads available.

The Iso-Seq Analysis pipeline also performs error correction through its ICE (Iterative Clustering and Error correction) algorithm; however, the resulting reads are consensus full-length isoforms (not CCS reads), which no longer include those non-full-length reads that we want to assemble.

Minimap2 was designed to handle the high rate of errors in long reads. We did an assessment on the accuracy of Minimap2 vs. GMAP on a set of datasets including both corrected and not-corrected ones. We found that Minimap2 is more accurate than GMAP for long reads without error correction, and Minimap2 and GMAP have nearly the same accuracy for long reads with error correction.

6. Line 16 Page 5, the authors say that "vertices are exons". As I know, each node in the graph is a continuous region in the reference genome. It is hard to say that each constructed vertex corresponds a real exon.

Thank you for your suggestion. We now have changed "vertices are exons" to “vertices are inferred (partial) exons” in the 1st paragraph of Section 2.1 in the manuscript.

7. I have a question that if there are some CCS reads mapped to multiple positions in the reference genome? If so, how do you deal with it?

If a CCS read is mapped to multiple positions in the reference genome, Minimap2 rates each of these multiple alignments by the score of the max-scoring sub-segment (excluding introns) and then marks the best alignment as primary in the SAM file. Minimap2 also reports up to 5 secondary alignments. Scallop-LR uses the best alignment (the primary in SAM) when building the splice graph.

**Reviewer #2 :**

General comments

The paper entitled “Quantifying the Benefit Offered by Transcript Assembly on Single-Molecule Long Reads” describes a novel algorithm for reference-based assembly of long error-prone RNA reads. The authors extend their previous short-read assembler Scallop by adding several modification, that allow to benefit from reads covering several splice junctions and “boundary reads” containing 5’ or/and 3’ primers. Since protocols for sequencing long RNA reads appeared rather recently, very few methods for its processing exist at the moment, so a high-quality long-read transcriptome assembler is a valuable instrument for the scientific community.

The authors use different existing methods for quality evaluation on multiple datasets against the best available tools for assembling long RNA reads. Presented benchmarks seem to be fair and strongly support Scallop-LR good performance in comparison to other tools. What I find important, is that the authors upload all scripts and instructions necessary for experiment reproduction, something that should become a standard for all “tool papers”. However, I encountered some issues while running the analysis (see comments below).

The tool itself is available, documented and working, which, indeed, adds value to this work. Some dependencies (like Iso-seq pipeline) are a bit heavy, but looks like there is no other choice so far.

Overall, the paper is well-structured and relatively easy to follow. Below I summarize a few non-crucial issues and questions, which, in my opinion, may improve the quality of the current Manuscript.

Thank you for your positive comments.

Major comments

● Since the novel method is entirely based on the previous tool Scallop, the manuscript includes the description only for the key modifications introduced in Scallop-LR. Indeed, understanding of some novel features would require complete description of the previous algorithm. For example, it is not entirely clear how exactly phasing paths are preserved by the graph decomposition algorithm. Presenting the entire previous algorithm in detail is probably unnecessary, but I feel like section 2.1 would significantly benefit from a figure showing some simple example of a splice graph and its decomposition without and with long reads. Some algorithmic details in the supplementary material may also be an option.

Thank you for your suggestion. We have now added a figure for Section 2.1.1 showing a simple example of a splice graph and its decomposition without and with preservation of long reads’ phasing paths.

● Although it seems intuitive that Scallop-LR should outperform original Scallop, explicitly showing quality increase could be a significant justification for added algorithms.

Thank you for your suggestion. We have now compared the performance of Scallop-LR (v0.9.1) with the performance of Scallop (v0.10.3), and the results are shown in Supplementary Table S10. We also discussed these results in the Discussion section.

The precision of Scallop-LR significantly increases compared with that of Scallop. The sensitivity of Scallop-LR also increases compared with that of Scallop (except for two datasets Scallop has slightly higher sensitivity than Scallop-LR). These results show the benefits of the long-read-specific optimizations added in Scallop-LR.

● As one of the quality metrics, the authors use the number of x-y% matched transcripts. However, this metric is not normalized anyhow. For example, an assembler that simply duplicates every assembled transcript would have twice more matched transcripts. Worth noting, that Scallop-LR typically has fewer transcripts in its assembly than StringTie, but still outperforms it by the number of x-y% matched transcripts. Thus, normalization of this metric would not change the claim that Scallop-LR outperforms StringTie according to this parameter.

We agree that if an assembler outputs duplicated transcripts, using a normalized metric of “x-y% matched transcripts” would make more sense. However, our Gffcompare results reported that none of Scallop-LR, StringTie, and Iso-Seq Analysis outputs duplicated transcripts for the analyzed datasets. In this case, we think the number of “x-y% matched transcripts” would reflect the assembly outcome better--- outputting a larger number of nearly-matched transcripts would be more useful when there are no duplicated or redundant transcripts.

If we use a normalized metric of “x-y% matched transcripts” (e.g. dividing the number of “x-y% matched transcripts” by the total number of assembled transcripts), the denominator would play an important role. Iso-Seq Analysis outputs significantly fewer transcripts in total than Scallop-LR since it does not assemble non-full-length reads. Because of this significantly smaller denominator, although Iso-Seq Analysis has a much smaller number of “x-y% matched transcripts” in the 95-100% bin, it has a higher value of normalized “x-y% matched transcripts” in the 95-100% bin than Scallop-LR. This is consistent with the results that Iso-Seq Analysis has lower sensitivity and higher precision than Scallop-LR. Using the normalized metric of “x-y% matched transcripts” would be analogous to using precision, and using the number of “x-y% matched transcripts” would be analogous to using sensitivity (when there are no duplicated or redundant transcripts).

We agree that when there are no duplicated transcripts, if an assembler outputs nearly redundant (very similar but not the same) transcripts, the number of “x-y% matched transcripts” could also be artificially inflated. However, Scallop-LR uses a post-assembly clustering algorithm to remove those nearly redundant transcripts. Iso-Seq Analysis also removes nearly redundant transcripts as stated in its documentation. StringTie was originally designed as a short-read assembler and thus did not incorporate this kind of clustering. Therefore, StringTie has the lowest precision. Besides the ICE error correction and the polishing with Quiver, an important contributing factor to Iso-Seq Analysis’s higher precision is that it does not assemble non-full-length reads and thus outputs significantly fewer transcripts in total than Scallop-LR.

Additionally, Scallop-LR outputs many more “novel isoforms in catalog” than Iso-Seq Analysis (as identified by SQANTI). This indicates that many of Scallop-LR’s lower-%-matching transcripts (which increases the denominator in calculating precision or in calculating the normalized “x-y% matched transcripts” in the 95-100% bin) are very likely novel isoforms.

Because of these reasons, we think using the number of “x-y% matched transcripts” would reflect the assembly outcome better, although admittedly for StringTie this measure may be somewhat inflated.

● As stated in the paper, according to a very important metric “x-y% assembled isoforms” StringTie typically has a better assembly than Scallop-LR. It would be useful to check whether StringTie really assembles more isoforms, or it assembles some transcripts that are not in the reads, but were assembled by mistake. To check it, I propose to use simulated data, so that the initial set of expressed isoforms is known (ground truth). Thus, it would be possible to compute the percentage of x-y% assembled isoforms relative to this initial set, rather than all known isoforms. It is also possible to compute the number of isoforms that were falsely assembled (e.g. absent in the simulated data). Based on the statistics presented in the manuscripts, my guess it that StringTie may generate a significant number of falsely assembled isoforms.

By the way, comparison on simulated data can be useful regardless of the comment above.

Thank you for your suggestion. We now have used a simulated human dataset generated by the PacBio PBSIM tool to evaluate StringTie and Scallop-LR using rnaQUAST. For StringTie and Scallop-LR, the “x-y% assembled isoforms” relative to the initial set of expressed isoforms that was used to generate this simulated dataset are reported in Supplementary Table S13. The Gffcompare evaluation results for Scallop-LR and StringTie on this simulated dataset are reported in Supplementary Table S12. We discussed these results in Section 4 of the Supplementary Materials.

Scallop-LR has both higher sensitivity and higher precision than StringTie, consistent with the trends on the real human datasets. Scallop-LR assembles more correctly predicted transcripts than StringTie for the simulated dataset (Table S12). Note that since the simulated CCS reads do not contain the primer information, Scallop-LR’s transcript boundary identification through extracting the boundary information from long reads is not used on the simulated data. For the simulated dataset, StringTie has more “x-y% assembled isoforms” in the 95-100% bin than Scallop-LR (Table S13), consistent with the trend on the majority of the real human datasets. This result indicates that StringTie indeed assembles more “95-100% assembled isoforms” than Scallop-LR, rather than getting a higher value because of assembling many transcripts that are not in the reads but match the reference transcriptome by mistake, since now the metric “x-y% assembled isoforms” is computed relative to the initial set of expressed isoforms that was used to generate the simulated reads, rather than all known isoforms. This suggests that while Scallop-LR outperforms StringTie in terms of 100% assembled correct predictions (as reported by Gffcompare), StringTie transcripts cover more reference transcripts on 95-99% of their bases than Scallop-LR transcripts.

We further performed the rnaQUAST evaluation on the simulated dataset by using the entire Ensembl annotation (Homo sapiens GRCh38.94) as the reference annotation. The resulting “95-100% assembled isoforms” are 3094 and 4613 for Scallop-LR and StringTie respectively. Compared with the corresponding results in Table S13 (which uses the “ground truth” as the reference annotation), StringTie assembles 365 more “95-100% assembled isoforms” when the entire Ensembl annotation is used as the reference. This implies that StringTie assembles 365 transcripts that are not in the ground truth but appear to be misassembled to match the reference transcriptome. For Scallop-LR, the number of “95-100% assembled isoforms” by using the entire Ensembl annotation as the reference is very close to the number of “95-100% assembled isoforms” by using the “ground truth” as the reference (the difference between the numbers of “95-100% assembled isoforms” by using these two references is -9). This result may suggest that, for the real datasets, the “95-100% assembled isoforms” of StringTie could be somewhat inflated (i.e. some assembled transcripts are not in the ground truth but are misassembled to match certain transcripts in the reference), as we do not know the ground truth for the real datasets and use all known isoforms as the reference when evaluating the real data. On the other hand, based on this evidence, it seems that Scallop-LR stays consistent on this measure when the entire Ensembl annotation or the “ground truth” is used as the reference.

● Although it took some time, I reproduced the experiments according to instructions here https://github.com/Kingsford-Group/lrassemblyanalysis . Eventually, I managed to get to the Scallop-LR assembly step. I’d suggest the following:

○ Give more details on how to prepare the reference data (e.g. gmap db and gmap reference sets).

Thank you for your suggestion. We now added descriptions on how to prepare gmap db and gmap reference sets in the README page of the above GitHub repository.

○ Since paths to the reference data are neither provided as parameters nor set in some config file (which would be more convenient), adding details on which lines of code should be changed could ease the process. Looking through the code or error logs can be a bit time consuming.

For the paths to the reference data, we now marked the lines of code that need to be changed by “# REPLACE WITH YOUR ACTUAL PATH TO THE REFERENCE DATA” in the scripts.

○ Some binaries are expected to be in $PATH (like minimap2), some have hardcoded local path in the code (Scallop, Gffcompare).

We now made it so that no binaries have local paths in the scripts. Now, all binaries are expected to be in $PATH. We also updated the instructions in the README page accordingly.

○ Bioawk dependency is not specified.

We now added bioawk in the tools/programs table in the README page of the above GitHub repository.

○ Re-running script biosample_isoseq.sh after a failure gives error messages (tries to create existing directory or links). Not critical, but extra error messages do confuse.

In the biosample_isoseq.sh script, we now made the soft link creation to still work if the link already exists. If the analysis output directory already exists with partial or corrupted data due to a previous failure run, we now clean up that directory first in the script.

○ Scripts are not compatible with newer smrtlink versions. A note on that can also be added.

We now added a note that the scripts for Iso-Seq Analysis are for SMRT Link v5.1.0 and are not compatible with later SMRT Link versions in the README page of the above GitHub repository.

Minor comments

● While the term “assembly” relates to two different groups of methods — de novo and reference-based (probably even more often to de novo), I suggest to mention that the developed method is reference-based as early as possible, e.g. in the beginning of the introduction or even in the abstract.

Thank you for your suggestion. We now added “reference-based” earlier in the Introduction (i.e. Background) where Scallop-LR is first introduced. We also added “reference-based” in the Abstract.

● Section 2.1.2. It is not clear where variables c and d come from. Are those just notations for arbitrary exons starting at position a (ending at position b)?

We have now made it clear what variables c and d are in Section 2.1.2.

● Section 2.1.2. Let’s say the allowed variance for transcript start discovery is 5 bp, and we have six 5’ boundary reads aligned to positions [95, x1 ], [98, x2 ], [101, x3 ], [105, x4 ], [107, x5 ], [110, x6 ]. In this case, we can pick, for example, position 105 as a starting position of a transcript, or we can pick two alternative starts 98 and 107 (both supported by 3 reads within variance region). How would algorithm work in this case?

Scallop-LR first sorts all boundary positions (from boundary reads) together with splicing positions. Scallop-LR identifies a new transcript boundary if the number of adjacent boundary positions of the same type (i.e., not separated by any different type of boundary or splice position in the sorted list) reaches a threshold (by default 3). In the above example, if these 6 positions are adjacent in the sorted list then Scallop-LR regards them as one 5’ boundary and reports the left-most one (i.e. 95 in this example) as the 5’ transcript boundary coordinate (if they are 3’, then the right-most one will be used).

● The authors are honest about lower precision on large datasets for Scallop-LR. It would be interesting to understand the reason of such behaviour. Could it be the effect of using some absolute thresholds in the algorithm?

As the sequencing depth goes up in large datasets , more lowly-expressed transcripts can be captured by RNA-seq reads. Thus, Scallop-LR is able to identify more such lowly-expressed transcripts (Tables S2 and S5 show that Scallop-LR finds many more potential novel isoforms in those much larger datasets ), as its core algorithm can preserve all phasing paths (the Scallop paper illustrated the improvement of Scallop over other methods in assembling lowly-expressed transcripts). However, overall lowly-expressed transcripts are harder to assemble (as transcripts may not be fully covered by reads), which may lead to the relatively lower precision on large datasets. Assembling more potential novel isoforms would also lower the precision on large datasets as the precision is computed based on the predicted known transcripts.

● It may be useful to mention that an unannotated transcript reported by rnaQUAST denotes an assembled transcripts mapped to intergenic space, and thus does not relate to the number of novel isoforms discovered by other methods. In my opinion, it may possibly be confusing for a reader why the number of novel isoforms is significantly larger than the number of unannotated transcripts, but I leave this decision to the authors.

Thank you for your suggestion. In the 4th last paragraph in Section 3.4, we now added that “an unannotated transcript reported by rnaQUAST denotes an assembled transcript mapped to intergenic space, and thus does not relate to the novel isoforms identified by Gffcompare or SQANTI”.

● Although authors mention TransRate, it remains unclear how exactly it was used in quality evaluation.

We use TransRate to obtain statistics of predicted transcripts including the minimum, maximum, and mean lengths, the number of bases in the assembly, and numbers of transcripts in different size ranges (the 2nd last paragraph in Section 2.2). TransRate also has a reference-free evaluation module which aligns the reads used in the assembly to the assembled contigs to evaluate each contig in the assembly. Unfortunately this reference-free evaluation module only works for paired-end reads and does not support single-end reads. So we cannot use this module for long reads since long reads are single-ended.

● GitHub repository does not contain any information on Scallop-LR and its pipeline (except the release archives).

We have now added a README page in the Scallop-LR GitHub repository to introduce the installation and usage of Scallop-LR, available at:

<https://github.com/Kingsford-Group/scallop/tree/isoseq>

**2^nd^ round**

**Reviewer 1**

The authors revised the manuscript and answered the questions I posed.

**Reviewer 2**

The authors did a great job answering all questions and resolving all issues, including minor one. I have no further comments. Congratulation on a great tool!

Due to my support of open peer review policy, I hereby sign my response.

Sincerely yours

Andrey Prjibelski
